# High expression of integrin *β*6 in association with the Rho–Rac pathway identifies a poor prognostic subgroup within HER2 amplified breast cancers

**DOI:** 10.1002/cam4.756

**Published:** 2016-05-17

**Authors:** Krisha Desai, Madhumathy G. Nair, Jyothi S. Prabhu, Anupama Vinod, Aruna Korlimarla, Savitha Rajarajan, Radhika Aiyappa, Rohini S. Kaluve, Annie Alexander, P. S. Hari, Geetashree Mukherjee, Rekha V. Kumar, Suraj Manjunath, Marjorrie Correa, B. S. Srinath, Shekhar Patil, M. S. N. Prasad, K. S. Gopinath, Raman N. Rao, Shelia M. Violette, Paul H. Weinreb, T. S. Sridhar

**Affiliations:** ^1^ Division of Molecular MedicineSt. John's Research InstituteBangaloreIndia; ^2^ Kidwai Medical Institute of OncologyBangaloreIndia; ^3^ St. John's Medical College and HospitalBangaloreIndia; ^4^ Shankara Cancer Hospital and Research CentreBangaloreIndia; ^5^ Rangadore Memorial HospitalBangaloreIndia; ^6^ Biogen Inc.CambridgeMA

**Keywords:** Breast cancer, HER2 subtype, integrin *α*v*β*6, invasion, MMP, Rho‐Rac

## Abstract

Integrin *α*v*β*6 is involved in the transition from ductal carcinoma *in situ* (DCIS) to invasive ductal carcinoma (IDC) of the breast. In addition, integrin *β*6 (ITGB6) is of prognostic value in invasive breast cancers, particularly in HER2+ subtype. However, pathways mediating the activity of integrin *α*v*β*6 in clinical progression of invasive breast cancers need further elucidation. We have examined human breast cancer specimens (*N* = 460) for the expression of integrin *β*6 (ITGB6) mRNA by qPCR. In addition, we have examined a subset (*N* = 147) for the expression of *α*v*β*6 integrin by immunohistochemistry (IHC). The expression levels of members of Rho–Rac pathway including downstream genes (*ACTR2*,*ACTR3*) and effector proteinases (*MMP9*,*MMP15*) were estimated by qPCR in the HER2+ subset (*N* = 59). There is a significant increase in the mean expression of ITGB6 in HER2+ tumors compared to HR+HER2‐ and triple negative (TNBC) subtypes (*P* = 0.00). HER2+ tumors with the highest levels (top quartile) of ITGB6 have significantly elevated levels of all the genes of the Rho–Rac pathway (*P*‐values from 0.01 to 0.0001). Patients in this group have a significantly shorter disease‐free survival compared to the group with lower ITGB6 levels (HR = 2.9 (0.9–8.9), *P* = 0.05). The mean level of ITGB6 expression is increased further in lymph node‐positive tumors. The increased regional and distant metastasis observed in HER2+ tumors with high levels of ITGB6 might be mediated by the canonical Rho–Rac pathway through increased expression of MMP9 and MMP15.

## Introduction

The expression of integrin *α*v*β*6 (ITGB6) is largely restricted to developing tissues, wound‐healing, and malignant epithelia and is not usually seen in the normal epithelia 1, 2, 3, 4. There is now overwhelming evidence for its role in the clinical progression of multiple human epithelial malignancies (colon, ovary, and brain), both from the *in situ* to the invasive stage as well as from the invasive stage to regional and distant metastasis 5, 6, 7. The addition of breast cancers to this long list of epithelial cancers where integrin *α*v*β*6 plays a role in clinical progression is more recent. Presence of integrin *β*6 on myoepithelial cells has been shown to be correlated with the progression from ductal carcinoma *in situ* (DCIS) to invasive ductal carcinoma (IDC) by generating a tumor promoter function through up‐regulation of matrix metallo‐protease 9 (MMP9) 8, 9. Within human breast cancers, the subset that have overexpression of the growth factor receptor HER2, have been shown to have the highest levels of integrin *α*v*β*6. Tumors with overexpression of HER2 tend to have a poor prognosis in general 10. In most populations, approximately 20% of the breast cancer patients meet the diagnostic thresholds set for positive expression of HER2 protein 11, 12.

There are currently no clinically validated markers that can effectively prognosticate the good from the poor outcome classes within the HER2+ subtype. Since almost all these tumors are highly proliferative, the differences in clinical outcomes in this subtype might arise from other attributes, such as responsiveness to chemotherapy and targeted therapy, or the propensity to metastasize. An established means of tumor metastasis is through altered tumor‐epithelial‐stromal interactions. In an evolving tumor, the stromal cues bring about changes in the extracellular matrix leading to activation of a family of integrins that mediate attachment of tumor cells to the extracellular matrix (ECM) 13. Components of the ECM, like fibronectin and tenascin and the latency‐associated peptide 1 and 3 have been shown to activate *α*v*β*6 integrin leading to local activation of TGF*β*1 signaling at tumor‐stroma interface facilitating tumor migration and invasion 14, 15, 16, 17.

Physical interaction between integrins and several oncogenes and receptor tyrosine kinases (RTKs) like HER2 and EGFR leads to integrated signaling cascades that have been implicated in tumor progression 18, 19, 20. A recent study carried out by Moore et al. has reported the involvement of *α*v*β*6 in metastatic progression of HER2 overexpressing breast tumors 14. This cooperation between integrins and RTKs leads to activation of the canonical Rho–Rac pathway through a large number of mechanisms. Pioneering work of Alan Hall has described the role of Rho GTPases in regulating assembly of focal adhesions in response to growth factors leading to increased cell migration 21. Activated Rho GTPases like RAC1, CDC42, and RHOA through kinases like p‐21 activated kinase (PAK) and PI3K control cell shape by bringing about changes in the actin stress fibers and polymerization of F‐actin through ACTR2/3 complex resulting in cell spreading and migration 22, 23.

In this study, we have confirmed the prognostic importance of integrin *β*6 in invasive breast tumors of the HER2 subtype, where the association is strongest. More importantly, we provide evidence for the increased expression of members of the Rho–Rac pathway in HER2+ tumors with the highest expression (top quartile) of ITGB6. Apriori identification of this subgroup in the clinical setting might enable testing of the efficacy of anti‐integrin therapy as an addition to the proven anti‐HER2 therapies.

## Methods

**Figure 1:**
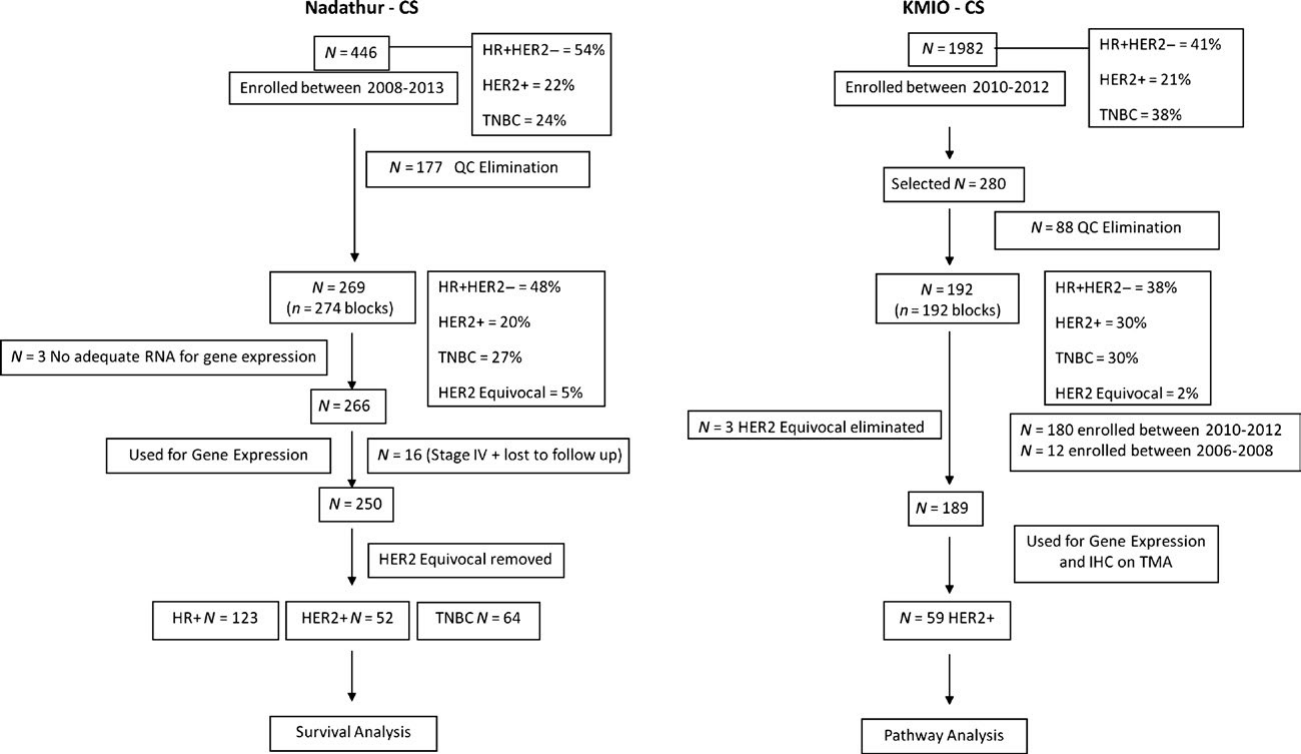
Flow chart depicting utilization of tumors from the two series. HR+, hormone receptor positive; TNBC, triple negative; QC, quality control.

Tumor samples from surgically excised breast tumors were selected from two non‐consecutive case series. In the first series called the Nadathur Case Series (Nadathur‐CS), 446 patients were enrolled prospectively at two tertiary‐care hospitals (St. John's Medical College and Hospital and Sri Rangadore Hospital) in Bangalore, from June 2008 to February 2013. The other case series is from the Kidwai Memorial Institute of Oncology (KMIO), a regional cancer center, wherein tumor blocks of 280 patients from 1982 breast cancer specimens examined at the department of pathology between 2010 and 2012 were collected from the archives. The choice of these 280 patient blocks was based on accessibility and availability, except for the choice of the HER2+ blocks which were ones that had been segregated earlier. In the Nadathur‐CS on which the survival analysis was performed, the set used for molecular analysis resembles the larger set of 446 tumors in almost all of the important clinicopathological parameters as shown in Table S1. Informed consent for use of the material for research was obtained from all patients in the Nadathur‐CS and the study was approved by the IERB (Institutional Ethics Review Board) at all hospitals, including KMIO. Samples were fixed in 10% neutral buffered formalin at room temperature (RT) and stored as formalin fixed paraffin‐embedded blocks (FFPE). All tissues were sectioned and stained with hematoxylin and eosin (H&E, Merck, Darmstadt, Germany). Only those with >50% tumor content as estimated by a pathologist (J.S.P.) were chosen for analysis. Clinicopathological characteristics like age, type of tumor, grade, and lymph node status along with ER, PR, and HER2 status were obtained from the clinical and histopathological records for the two case series are listed in Table 1. This is a retrospective study and for the KMIO‐CS, all the information was obtained from the pathological records which did not have all the information needed for clinical staging. The details of the sequential exclusion of the tumor samples from the two case series for various analyses are outlined in Figure 1.

**Table 1:** Clinicopathological characteristics of cases from Nadathur and KMIO‐CS

	Nadathur ‐ CS	Nadathur ‐ CS	KMIO ‐ CS	KMIO ‐ CS
	All *N* (%) (*N* = 269 patients)	HER2+ *N* (%) (*N* = 53 patients)	All *N* (%) (*N* = 192 patients)	HER2+ *N* (%) (*N* = 59 patients)
Age				
Mean	56	55	49	46
Median	56	55	48	45
Tumor size				
T1	72 (27)	14 (26)	28 (15)	9 (15)
T2	160 (59)	32 (60)	140 (73)	41 (69)
T3	28 (10)	6 (11)	21 (11)	8 (14)
Tx	9 (3)	1 (2)	3 (2)	1 (2)
Lymph node status				
N0	112 (42)	20 (38)	61 (32)	14 (24)
N1	81 (30)	18 (34)	21 (11)	6 (10)
N2	40 (15)	8 (15)	44 (23)	14 (24)
N3	27 (10)	7 (13)	53 (28)	21 (36)
Nx	9 (3)	0	13 (7)	4 (7)
	**(** ***n*** ** = 274 blocks)**		**(** ***n*** ** = 192 blocks)**	
Grade				
I	19 (7)	1 (2)	2 (1)	Nil
II	126 (46)	25 (47)	31 (16)	5 (8)
III	113 (41)	26 (49)	153 (80)	53 (90)
Nx	16 (6)	1 (2)	6 (3)	1 (2)
Stage				
I	42 (16)	7 (13)		
II	134 (50)	25 (47)		
III	83 (31)	20 (38)		
IV	10 (4)	1 (2)		
Menopausal status				
Pre	74 (27)	17 (32)	81 (42)	29 (49)
Post	200 (73)	36 (68)	65 (34)	18 (31)
Unknown		Nil	46 (24)	12 (20)
Estrogen receptor				
Positive	160 (58)	19 (36)	95 (49)	20 (34)
Negative	114 (42)	34 (64)	97 (51)	39 (66)
Progesterone receptor				
Positive	103 (38)	16 (30)	57 (30)	9 (15)
Negative	171 (62)	37 (70)	135 (70)	50 (85)
HER2				
Positive	53 (19)		59 (31)	
Negative	207 (76)		130 (68)	
Equivocal	14 (5)		3 (2)	

In the KMIO‐CS, blocks from 192 patients of the 280 screened, passed all QC criteria including molecular criteria. Three HER2 equivocal samples were eliminated from the analysis, restricting it to 189 patients.

In the Nadathur‐CS, of the 274 blocks that met QC criteria for molecular analysis from 269 patients, 31 cases were assigned to the HER2 equivocal class by IHC criteria. These blocks were retested by fluorescent in situ hybridization (FISH). Seventeen of the 31 cases did not meet the FISH criterion of positivity and were classified as HER2 negative. Fourteen of the 31 could not be tested for technical reasons and hence, remained as HER2 equivocal. All 266 cases were used for gene expression as three cases did not have enough tissue to assay for ITGB6 expression. Of the 266, 11 cases had metastasis at presentation, two were lost to follow‐up, and three cases could not be staged unambiguously resulting in 250 patients who had complete information and were considered for survival analysis. Of the 53 HER2+ patients, one had distant metastasis at presentation and therefore, only 52 were considered for survival analysis. The median follow‐up of all 250 patients was 59 months, and for the 52 HER2+ patients it was 62 months as of 31 December 2015. The treatment history is provided in the supplementary data.

### FISH for HER2 gene amplification

Five micrometer sections were cut on to PLL coated slides and incubated at 56°C overnight. Slides were deparaffinized in two changes of xylene for 30 min each followed by dehydration in graded alcohol. Slides were denatured in 2X saline‐sodium citrate (SSC) at 75°C for 20 min followed by digestion with proteinase K (0.01 *μ*g in 10% sodium lauryl sulfate) for 10 min for optimum digestion. Subsequently, slides were incubated with 4 *μ*L of HER2 probe (HER2/Neu labeled with PlatinumBright 550 and SE 17 control DNA probe labeled with PlatinumBright 495, Poseidon probes, Kreatech Diagnostics, Amsterdam, Netherlands) in a humidified chamber for 17 h. Post hybridization wash was done in 2X SSC with 0.3% NP‐40 solution at 75°C for 3–4 min followed by a second wash in 2X SSC for 2 min at RT. Slides were further dehydrated, nucleus counterstained with DAPI and viewed through Olympus BX51 fluorescent microscope for HER2 and SE17 signals using the appropriate filter sets. Positive and negative controls were included in each batch for assay control and reproducibility. For scoring of the FISH assay, the number of signals for HER2 and SE17 was done in at least 50 tumor cell nuclei and the ratio of HER2/SE17 signal was then calculated. Ratios above 2.2 were taken as amplified.

### RNA extraction and cDNA synthesis

Total ribonucleic acid (RNA) was extracted according to instructions provided in a previously published paper 24. Briefly, two 20 *μ* FFPE sections from each tumor sample were deparaffinized at 90°C for 10 min and subjected to overnight digestion at 65°C with TE buffer containing 1% SDS and Proteinase K (Qiagen, Valencia, CA, USA). Total RNA was then extracted using TRI Reagent (Sigma‐Aldrich, St. Louis, MO, USA) according to manufacturer instructions. Quantitation of RNA was done using ribogreen dye (Quant–iT Ribogreen RNA assay kit; Invitrogen, Carlsbad, CA, USA) on a fluorescent microplate reader (M200‐Pro Infinite Series; Tecan, Mannedorf, Switzerland). A quantity of 500 ng of total RNA was then reverse transcribed into cDNA using the ABI high‐capacity cDNA archive kit (ABI, Carlsbad, CA, USA) as per the manufacturer's protocol.

### Quantitative real‐time PCR

The qRT‐PCR experiments were performed as reported in previous publication 24 and have been reported according to the minimum information guidelines (MIQE) for publication of qRT‐PCR experiments 25. Briefly, transcript levels were measured by qRT‐PCR in duplicates with 5 ng template for FFPE sections per reaction, using SYBR Green master mix (SYBR^R^ Premix Ex Taq^™^ II Tli RNaseH Plus; Takara Bio Inc., Mountain View, CA, USA) on the LightCycler 480 II (Roche Diagnostics, Indianapolis, IN, USA) with a 3 pmol/L concentration of forward and reverse primers making the reaction volume 10 *μ*L. Cycle Threshold (*C*_*T*_) values for the test gene were in turn normalized to the mean *C*_*T*_ value of the three reference genes—*ACTB, RPLP0*, and *PUM1* for each tumor sample; designated as ∆*C*_*T*_. Relative expression of test genes were calculated by the ∆*C*_*T*_ method, as the difference in quantification cycle (*C*_*T*_) of the test gene and the mean *C*_*T*_ of the reference genes [∆*C*_*T*_ = *C*_*T*_ (test gene) − *C*_*T*_ (reference gene mean)]. Normalized values are represented as relative normalized units (RNU), which are calculated as 15 − ∆*C*_*T*_. A one‐unit increase in relative normalized units is assumed to reflect a two‐fold increase of template; detailed description of which has been published 24. For the transcript levels of ITGB6 and for the genes assayed in the Rho–Rac pathway, an adjusted RNU (ARNU) was calculated by taking the difference between the least value and corresponding value from each sample. Precaution was taken while designing primers specific to the intron‐spanning region of the genes. Primers for all genes were designed using the software Primer3Plus and manufactured by Eurofins, Bangalore, India. The amplicon sizes ranged from 77 to 113 bp. The primer sequences and product length in base pairs (bp) for the genes tested are given in Table S2.

### Construction of a TMA from the KMIO‐CS

The tissue micro array (TMA) was constructed using the Quick Ray manual tissue microarrayer from Unitma Co. Ltd., Seoul, Korea. The master block was constructed using the manufacturer's instructions. A master block grid plan was made with adequate precautions to ensure accurate orientation and unambiguous specimen identification. Briefly, the tumor region of interest was identified in the reference H&E‐stained slide by a pathologist and marked with an oil pen. The marked tissue was extracted from the donor block by using the QuickRay^®^ needle. The extracted tissue was delivered into the appropriate hole of a pre‐made recipient block with 1.5 mm holes (UB06–1.5; 9 × 10 = 90 holes). The block was tapped to ensure that all tissue cores were at an even depth for best results when sectioned by microtomy. The recipient block was placed into the embedding mold with the cut section face down, and incubated at 70°C for 30–60 min, and removed when completely transparent. The block was then allowed to solidify in the cold. At least two cores were taken from each donor block. A cut section from the master block was stained with H&E and documented by photomicrography. TMA cores with less than 100 invasive tumor cells were considered inadequate for interpretation. Images of the H&E‐stained sections of a complete block (90 cores) and at a higher magnification, as well as images of IHC staining of multiple cores and a complete 1.5 mm core (1.75 sq. mm) are provided in Figure S2.

### Immunohistochemistry of TMA sections

IHC for *α*v*β*6 integrin was done on the tissue microarray built from KMIO‐CS. Briefly, sections (5 *μ*m in thickness) were cut from the master TMA block on to poly l‐lysine (PLL)‐coated slides and subjected to deparaffinization in xylene and rehydrated in graded alcohol. After blocking endogenous peroxidase with a 3% hydrogen peroxide solution, antigen retrieval was done in 0.01 mol/L EDTA buffer at pH 8 using a heat triggered MERS (multi‐epitope retrieval system, PathnSitu, Livermore, CA, USA) for 15 min at 90°C. Primary blocking was done with 1% bovine serum albumin (BSA, Sigma, St. Loius, MO, USA) for 30 min at room temperature. Primary antibody for *α*v*β*6, clone 6.2‐A1 (Biogen Inc., Cambridge, MA, USA) was used as previously described 26. The antibody was diluted to 0.17 *μ*g/mL and was applied for 1 h at room temperature. Sections were further incubated with secondary antibody (DAKO REAL^™^ EnVision^™^, Glostrup, Denmark) for 20 min as per the kit instructions, followed by development of the color using DAB (DAKO REAL^™^ EnVision^™^) for 10 min. Sections were counterstained with hematoxylin and mounted after dehydration in graded alcohol and xylene. Appropriate positive and negative controls were run for each batch. Scoring of integrin *α*v*β*6 was done as reported by Moore et al. 14 in their supplementary data. TMA cores with less than 100 invasive tumor cells were considered inadequate for interpretation and were dropped from further scoring. Integrin *α*v*β*6 staining was scored as the sum of the extent of tumor cells staining (0, <25% = 1, 25–50% = 2, 50–75% = 3, >75% = 4) and intensity (0 = negative, 1 = weak, 2 = moderate, 3 = strong); giving a final score range of 0–7. Each tumor core was scored by a pathologist; final score represents mean of two readings. Only 147/189 tumor cores were interpretable after the staining. A score of ≥6 was considered as unambiguous detection of the protein.

### Statistical analysis

To examine the concordance between ITGB6 transcript levels and the *α*v*β*6 protein levels, receiver operating characteristic (ROC) analysis was performed. Compared to the transcripts with higher abundance like *ESR1*, the level of expression of ITGB6 mRNA in the tumor samples was lower. Therefore, to study the biology mediated by ITGB6 in HER2+ tumors, we chose to select tumors with higher than the top quartile expression (cut‐off value of 9.22 for KMIO‐CS and 8.7 for Nadathur‐CS) of ITGB6 mRNA and grouped them as tumors with high ITGB6 expression (ITGB6‐H). Tumors below the top quartile were designated as ITGB6‐L. In general, all data were statistically analyzed using one‐way analysis of variance (ANOVA), post‐hoc Bonferroni's multiple comparison test, and two‐tailed Student's *t*‐test. Kaplan–Meier analysis was used to examine the estimated differences in disease‐free survival between the ITGB6‐H and ‐L groups across all tumor subtypes and within the HER2+ subtype. Disease‐free survival was calculated as the time from the date of first diagnosis to the time when either a local recurrence or a distant recurrence occurred. Patients without an event or had succumbed to non‐breast cancer related causes were right‐censored. Log‐rank test (Mantel‐Cox) was used to compare the survival between groups. Both univariate and multivariate Cox‐proportional hazard analysis were done to validate the prognostic importance of ITGB6 in comparison to other clinicopathological characteristics. For all tests, a *P*‐value of <0.05 was considered to be statistically significant. All statistical analysis were carried out using the software XLSTAT 2015 (Windows) and SPSS software version 18 (Chicago, IL).

## Results

### ITGB6 expression is associated with HER2 overexpressing tumors

**Figure 2:**
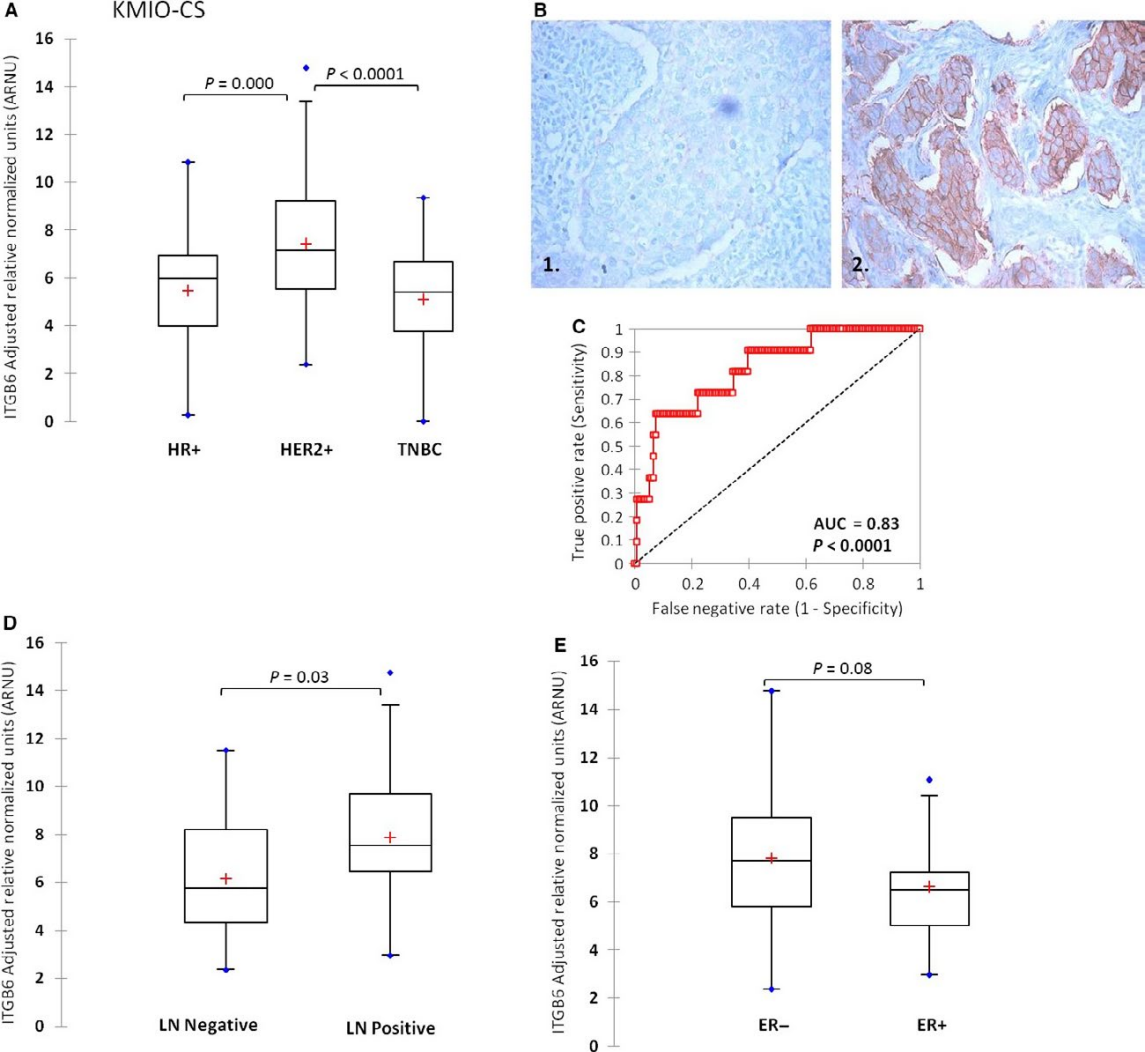
(A) Distribution of ITGB6 transcript in the KMIO‐CS divided into clinical subtypes. (B) Representative images of *α*v*β*6 staining in tumor sections. (1) 1+ intensity showing negative staining, (2) 3+ intensity showing a positively‐stained tumor section. (C) ROC analysis showing concordance between ITGB6 transcript and *α*v*β*6 protein. (D) Distribution of ITGB6 mRNA levels based on lymph node status (*P* = 0.03). (E) Distribution of ITGB6 mRNA in estrogen receptor groups (ER negative, ER−, ER positive, ER+).

We first assayed for the transcript level of integrin *β*6 in the KMIO–CS comprising of 189 breast tumors and found that its mean level was different between the clinical subtypes (ANOVA, *P* = 0.000). The expression was significantly higher in the HER2+ subtype (*N* = 59) in comparison to the HR+HER2‐ (*N* = 73) and TNBC subtypes (*N* = 57) as shown in Figure 2A. The dynamic range of ITGB6 mRNA was 14 C_T_ across all tumors. Next, we assayed for the presence of *α*v*β*6 by immunohistochemistry on TMA sections of the 189 tumors (147/189 tumor sections were interpretable). The scoring of the *α*v*β*6 IHC was done as described in Methods by an Allred‐like score (intensity and proportion of cells stained) producing a range of values from 0 to 7. A value of equal to and above 6 was considered as high protein expression. Thirteen percent of HER2+ tumors had high expression as opposed to 4% in HR+ and 6% in TNBC subtype. The representative images of TMA sections are shown in Figure 2B. The concordance between the mRNA and the protein was then investigated using ROC analysis by taking mRNA expression values as continuous distribution and *α*v*β*6 protein expression grouped into high and low, based on a cut‐off score of 6. We found a significant concordance (AUC of 0.83 (*P* < 0.0001)) with 0.63 sensitivity and 0.93 specificity; Figure 2C. Since, the detection at the transcript level is only for the *β*6 subunit of integrin and at protein it is for the hetero‐dimeric *α*v*β*6 integrin, we chose to take a cut‐off for ITGB6 mRNA above the top quartile (3rd quartile at 9.22), in order to maximize the specificity.

Among the classical traditional clinicopathological variables, involvement of the regional lymph nodes (LN) has the highest hazard for a poor clinical outcome. Hence, we compared the ITGB6 transcript levels between LN‐positive versus LN‐negative tumors of the HER2+ class. We found that mean ITGB6 mRNA level in those tumors that had metastasized to the lymph node (*N* = 44) was significantly greater (two‐tailed student's *t*‐test, *P* = 0.03) compared to the tumors that did not metastasize (*N* = 14). This suggests that ITGB6 expression is correlated with lymph node metastasis; Figure 2D.

A slightly greater proportion of HER2+ tumors tend to be estrogen receptor negative (ER‐) in most series. In the series of 59 HER2+ tumors, 39 were ER‐ and had a greater mean ITGB6 expression compared to HER2+ ER+ tumors (*N* = 20); two‐tailed student's *t*‐test, *P* = 0.08; Figure 2E.

### Proposed mechanism for ITGB6‐mediated metastasis in HER2‐positive tumors

**Figure 3:**
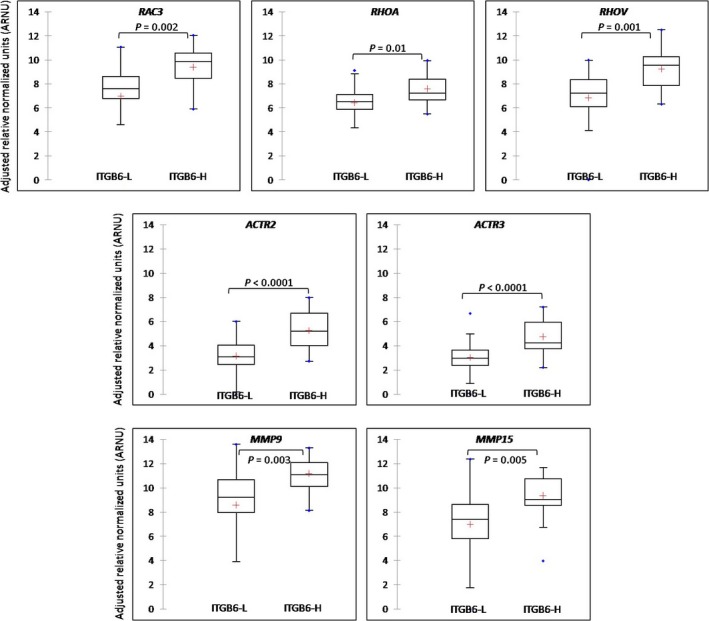
Differential expression of genes involved in the Rho–Rac pathway between ITGB6‐H and ‐L expressing tumors (two‐tailed student's *t*‐test, *P* < 0.05).

A large body of evidence exists for the integrin‐mediated cell migration and invasion through activation of the Rho–Rac pathway 27, 28. To look for the probability of such an association in the clinical specimens, we assayed for the transcript abundance of the genes involved in this pathway. We divided the 59 HER2+ tumors (as explained in materials and methods) into ITGB6‐H and ‐L groups based on the top quartile cut‐off value. Genes assayed involved—*RAC3*,* RHOA, RHOV*,* ACTR2* and *ACTR3*—genes that mediate lamellopodium extension by actin polymerization during migration and *MMP9* and *MMP15*—genes responsible for proteolytic cleavage of ECM proteins constituting the basement membrane leading to invasion. There was a significant positive correlation between ITGB6 mRNA and *RAC3* (*r* = 0.32), *RHOA* (*r* = 0.26), *RHOV* (*r* = 0.38), *ACTR2* (*r* = 0.42), *ACTR3* (*r* = 0.54), *MMP9* (*r* = 0.41), and *MMP15* (*r* = 0.44). The mean expression levels of all of these genes in the ITGB6‐H tumors was significantly higher (*P* < 0.05) compared to the ITGB6‐L tumors as shown in Figure 3. This association of ITGB6 mRNA with the genes involved in Rho‐Rac‐dependent migration and secretion of MMPs for ECM degradation is supported by other in vitro results using cell line models 9, 29, 30.

### ITGB6 as a prognostic marker in HER2‐positive tumors

**Figure 4:**
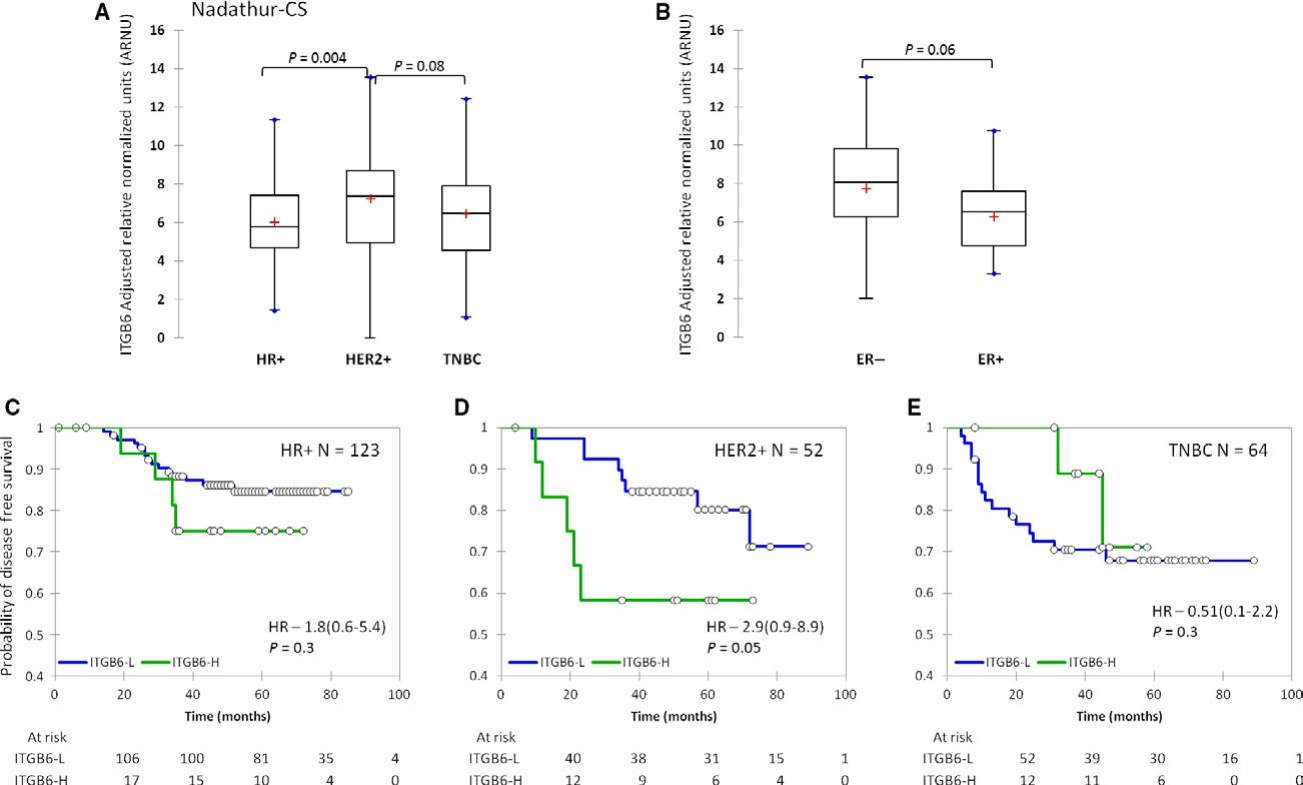
(A) Distribution of integrin *β*6 (ITGB6) mRNA expression in the three clinical subtypes of Nadathur‐CS. (B) Distribution of ITGB6 mRNA levels between estrogen receptor‐positive and ‐negative tumors within the HER2 subtype. (C, D, and E) Kaplan–Meier disease‐free survival between ITGB6‐H and ‐L tumors in HR+, HER2+, and TNBC subtype, respectively, blue line indicates low ITGB6 and green line indicates high ITGB6 expression.

In order to study the prognostic importance of ITGB6, we performed Kaplan–Meier survival analysis on the Nadathur‐CS set of samples. KMIO‐CS did not have survival information available and could not be a part of this analysis. We first validated the subtype specific expression of ITGB6 mRNA in this series of tumors and found that just as in the KMIO‐CS, the mean expression was different between the subtypes (ANOVA, *P* = 0.008). Also, the mean expression of ITGB6 was highest in the HER2+ subtype (*N* = 53), Figure 4A. A similar trend was also observed in this series for ITGB6 expression which tended to be higher in the tumors with metastasis to the regional lymph node (*N* = 34), *P* = 0.3 (data not shown). Also, HER2+ ER‐ tumors (*N* = 34) had a significantly higher mean expression of ITGB6 mRNA compared to HER2+ ER+ tumors (*N* = 19), *P* = 0.06, Figure 4B.

In their analysis, Moore et al. found that approximately 15–16% of the London and Nottingham cohort had detectable *α*v*β*6 integrin expression 14. Our ROC analysis of the KMIO‐CS of *α*v*β*6 integrin protein versus ITGB6 transcript levels had yielded a good concordance (AUC = 0.83; *P* < 0.0001) with 93% specificity. In order to maximize this specificity, we chose the cut‐off at 95% specificity (cut‐off of 9.5 for ITGB6 ARNU) and divided the samples into ITGB6‐H and ‐L. We then examined the 250 patients of the Nadathur‐CS using this bifurcation and found that about 13% (*N* = 32) of all patients had high ITGB6 mRNA expression. This reassured us that we were most probably working with very similar thresholds for ITGB6 detection. We found that stratification of the HER2+ tumor samples by ITGB6 mRNA levels produced significant separation of the groups based on disease‐free survival with a hazard ratio (HR) of 2.9 (0.9–8.9); log rank *P* = 0.05 (Fig. 4D). Within the HER2+ subtype the 5‐year probability of disease‐free survival dropped from 80% in ITGB6‐L tumors to 60% in ITGB6‐H tumors. However, ITGB6 mRNA could not separate disease‐free survival in the other clinical subtypes (Fig. 4C and E). The prognostic value of ITGB6 was also validated in the 52 HER2+ tumors using both univariate and multivariate Cox‐proportional hazard analysis, shown in Table 2. This data supports the contention for a role of integrin *β*6 in mediating metastasis in invasive breast cancers, particularly within the HER2 overexpressing tumors, suggesting a strong cooperation between the two in clinical progression of these tumors.

**Table 2:** Univariate and multivariate cox‐proportional hazard analysis

	Univariate		Multivariate	
	HR (95% CI)	*P*‐value	HR (95% CI)	*P*‐value
Age	1 (0.9–1.06)	0.8		
T ‐ size				
T1	Reference			
T2	0.5 (0.1–1.7)	0.2		
T3	0.9 (0.1–4.9)	0.9		
Lymph node				
Negative	Reference			
Positive	2.2 (0.6–8.2)	0.2		
Stage				
I	Reference			
II	0.7 (0.1–3.8)	0.7		
III	1.2 (0.2–6.3)	0.7		
Grade				
I	Reference			
II	0.7 (0.07–6.8)	0.7		
III	0.1 (0.01–1.7)	0.1		
Menopausal status				
Post	Reference			
Pre	0.8 (0.2–2.6)	0.7		
ER status				
Positive	Reference			
Negative	3.8 (0.8–17.4)	0.08	3.1 (0.6–14.9)	0.1
PR status				
Positive	Reference			
Negative	2.9 (0.6–13.2)	0.1		
ITGB6 RNU				
ITGB6‐L	Reference			
ITGB6‐H	2.9 (0.9–8.9)	**0.05**	2.1 (0.6‐6.8)	0.1

T‐size, tumor size; ER, estrogen receptor; PR, progesterone receptor. Statistically significant p‐value is in bold.

## Discussion

There is good evidence for the cooperative interaction between integrins and RTKs in neoplastic progression 18, 19. The role of Rho–Rac pathway in integrin‐mediated cell adhesion and migration has also been well studied. Integrins have been shown to promote actin assembly by recruiting molecules like Rac3 that activate actin polymerization and RhoA that increase contractility and transmit tension to the sites of integrin ligation leading to increased migratory potential 31, 32, 33. Also, integrin‐mediated production of matrix metalloproteases has been shown to aid tumor cells in degrading the basement matrix leading to invasion 9. Since, integrin engagement with ECM components has been shown to regulate the Rho‐Rac GTPases, we assayed for several crucial mediators of Rho‐Rac signaling cascade and found that they were highly correlated with HER2+ tumors possessing elevated ITGB6 transcript levels. We found a significant upregulation of each of these genes in the ITGB6‐H group. The mean mRNA expression of MMP9 and MMP15 was also significantly higher in ITGB6‐H tumors suggesting an integrin *β*6 mediated role in invasion. We validated this observation in 93 HER2+ tumors from the TCGA dataset by performing in‐silico data analysis and found significant upregulation of *RHOV* (*P* = 0.006), *RAC3* (0.05), *ACTR3* (*P* = 0.01), and *MMP15* (*P* < 0.0001) mRNA levels in ITGB6‐H tumors (Fig. S5). Since, multiple members of this pathway significantly correlate with ITGB6 mRNA expression, this association is suggestive of a potential role of coupled *α*v*β*6‐HER2 signaling in mediating migration and invasion of breast tumor cells through this pathway.

We observed that high ITGB6 expression was significantly correlated to the HER2+ clinical subtype and this coordinated expression of HER2 and ITGB6 was able to identify patients with decreased disease‐free survival. The results obtained from this study are consistent with the results reported by Moore et al. that signifies a role of *α*v*β*6 as an independent prognostic marker in HER2+ breast cancer. Their results suggest that simultaneous targeting of *α*v*β*6 with 264‐RAD antibody and of HER2 with Trastuzumab, significantly improves the therapeutic effect of trastuzumab alone and increases survival in xenograft models. This dual treatment may provide novel therapy for treating patients at high risk and with trastuzumab‐resistant breast cancer.

The data from this study also indicate that patients with high ITGB6 expression had significantly higher percentage of lymph node metastasis. This observation is in accordance with various studies that have reported the role of integrin *β*6 in mediating metastasis 15, 34, 35. However, as indicated on the graph (Fig. 2D), there was an overlap between the two groups with a subset of tumors with high ITGB6 expression with no lymph node metastasis and conversely, a few tumors with low ITGB6 expression had metastasized to the lymph node. Further, analysis of the TEX trial data 36 showed that HER2+ metastatic lesions possessed the highest mean expression of ITGB6 mRNA level (*P* < 0.0001, Fig. S3) compared to other PAM50 subtypes, further lending support to integrin *β*6‐mediated metastasis in HER2+ tumors. The in‐silico analysis of the 73 HER2+ cases obtained from the TCGA dataset (mentioned in Fig. S6) also showed significant positive correlation between ITGB6 mRNA and, as expected, ERBB2 (*r* = 0.56, *P* = 0.00) and EGFR (*r* = 0.33, *P* = 0.00) protein. A significant correlation between ITGB6 mRNA and Paxillin, a cytoskeletal protein involved in mediating cell adhesion to the ECM was also observed, (*r* = 0.27, *P* = 0.02) lending further support to the observations of cytoskeletal modulations in the setting of high ITGB6 expression.

Another interesting observation was the higher mean expression of ITGB6 mRNA in HER2+ tumors lacking the expression of ER protein. This class of tumors generally tends to have a poorer outcome with fairly aggressive biology. Due to the fewer numbers in this class, we were unable to probe this further. However, we validated this result by performing in‐silico TCGA analysis using a larger set of 93 HER2+ (Fig. S4). These observations warrant further investigation in a larger series of breast tumors. The intraclass variability within the HER2‐positive tumors (Figs. 2D, E and 4B) was observed to be much higher than the intersubtype variability (Figs. 2A, 4A) in ITGB6 expression suggesting that few tumors in the other subtypes also possess high levels of ITGB6 mRNA which would have led to a similar consequence in the presence of active growth factor receptor signaling.

## Conflict of Interest

None declared.

## Supporting information


**Table S1.** Clinicopathological characteristics of 446 and 269 patients in the Nadathur‐CS.

**Table S2.** Primer sequences used for gene expression analysis.

**Figure S1.** Distribution of ITGB6 mRNA in the two case series—KMIO and Nadathur‐CS. The dynamic ranges of ITGB6 ARNU in both the CS were similar and ranged from 0 to 14. ITGB6 mRNA expression followed a normal distribution in both the case series.

**Figure S2**. Representative tissue microarray sections. (A) Images of the Hematoxylin & Eosin‐stained sections of a complete block (90 cores) and at a higher magnification, (B) immunohistochemistry staining of integrin *α*v*β*6 on multiple TMA cores and a complete 1.5 mm core (1.75 sq. mm). TMA cores with less than 100 invasive tumor cells were considered inadequate for interpretation. A total of 189 tumor samples from the KMIO‐CS were used for building the TMA for *α*v*β*6 IHC staining and among them 147 were interpretable.

**Figure S3.** In‐silico data analysis from the TEX trial dataset. The distribution of ITGB6 transcripts was plotted across the PAM50 subtypes and intergroup variability was analyzed using the Kruskal–Wallis test. A *P*‐value of <0.05 was considered significant.

**Figure S4.** Data analyzed from 93 HER2+ tumors from TCGA dataset with ITGB6 mRNA information. ITGB6 mRNA distribution between ER− and ER+ groups, a *P*‐value of <0.05 was considered statistically significant.

**Figure S5.** Data analyzed from 93 HER2+ tumors from TCGA dataset with mRNA information available. Distribution of *RHOV, RAC3,* and *MMP15* between ITGB6‐L and –H groups.

**Figure S6.** Data analyzed from 73 HER2+ tumors from TCGA dataset with both ITGB6 mRNA and RPPA data available. Correlation plots between ITGB6 mRNA and ERBB2, EGFR, and PXN proteins. Pearson's correlation coefficient, r indicates the strength of correlation and a *P*‐value of <0.05 was considered statistically significant.
