# Supplementary figures and images for: High expression of integrin β6 in association with the Rho–Rac pathway identifies a poor prognostic subgroup within HER2 amplified breast cancers
Source: Cancer Med. 2016 May 17;5(8):2000–11. doi: 10.1002/cam4.756 (PMC4873607; doi:10.1002/cam4.756)

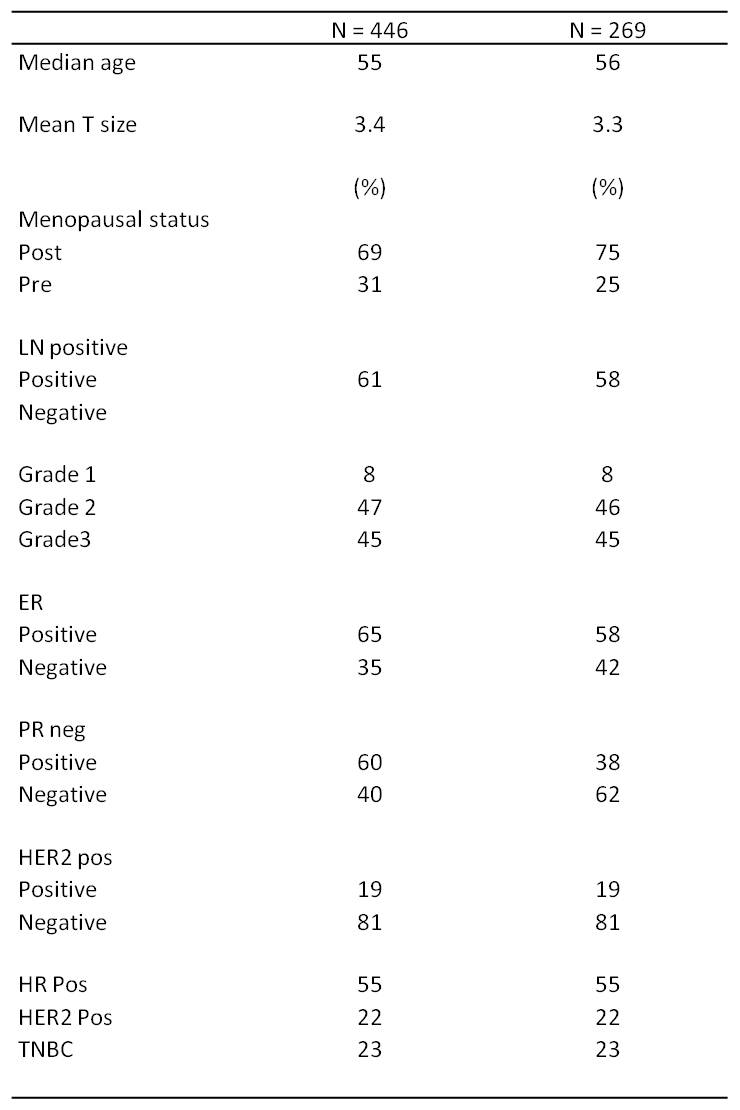

Supplement: Supplementary file 1 — Table S1. Clinicopathological characteristics of 446 and 269 patients in the Nadathur‐CS. [file CAM4-5-2000-s001.tif]

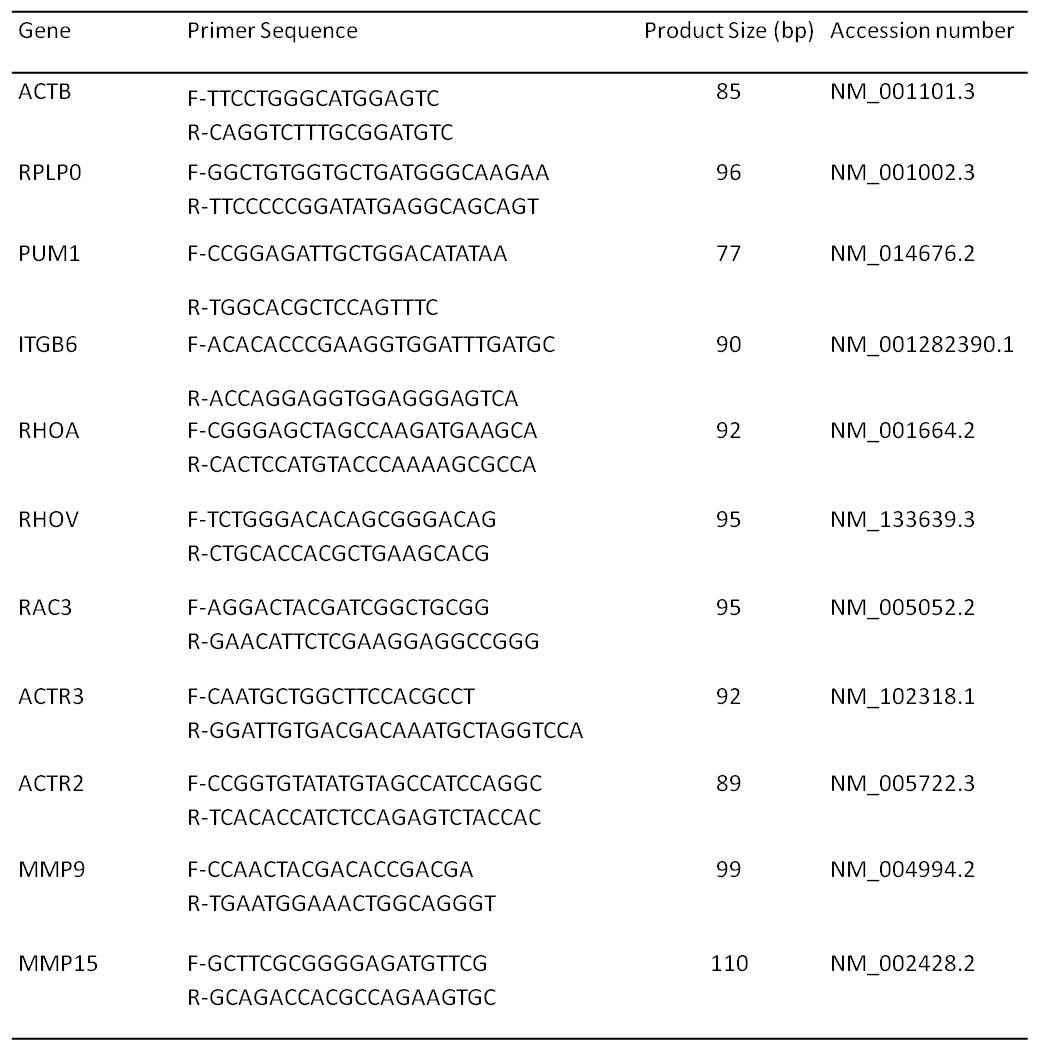

Supplement: Supplementary file 2 — Table S2. Primer sequences used for gene expression analysis. [file CAM4-5-2000-s002.tif]

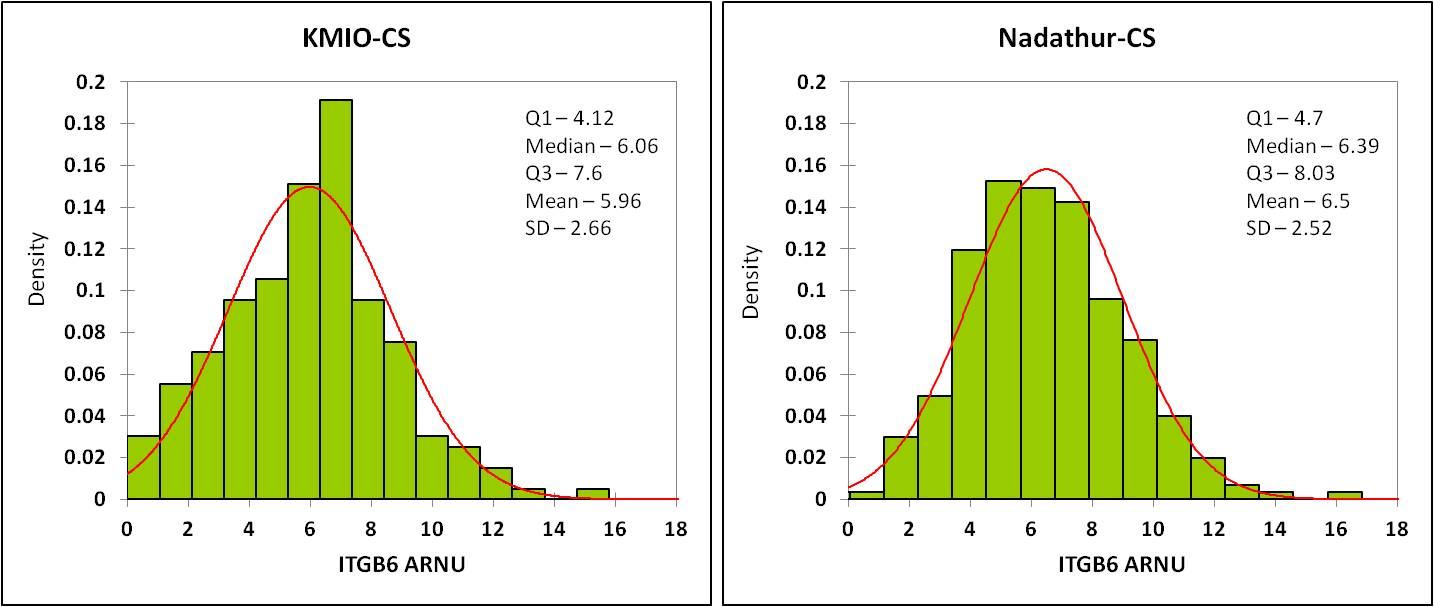

Supplement: Supplementary file 3 — Figure S1. Distribution of ITGB6 mRNA in the two case series—KMIO and Nadathur‐CS. The dynamic ranges of ITGB6 ARNU in both the CS were similar and ranged from 0 to 14. ITGB6 mRNA expression followed a normal distribution in both the case series. [file CAM4-5-2000-s003.tif]

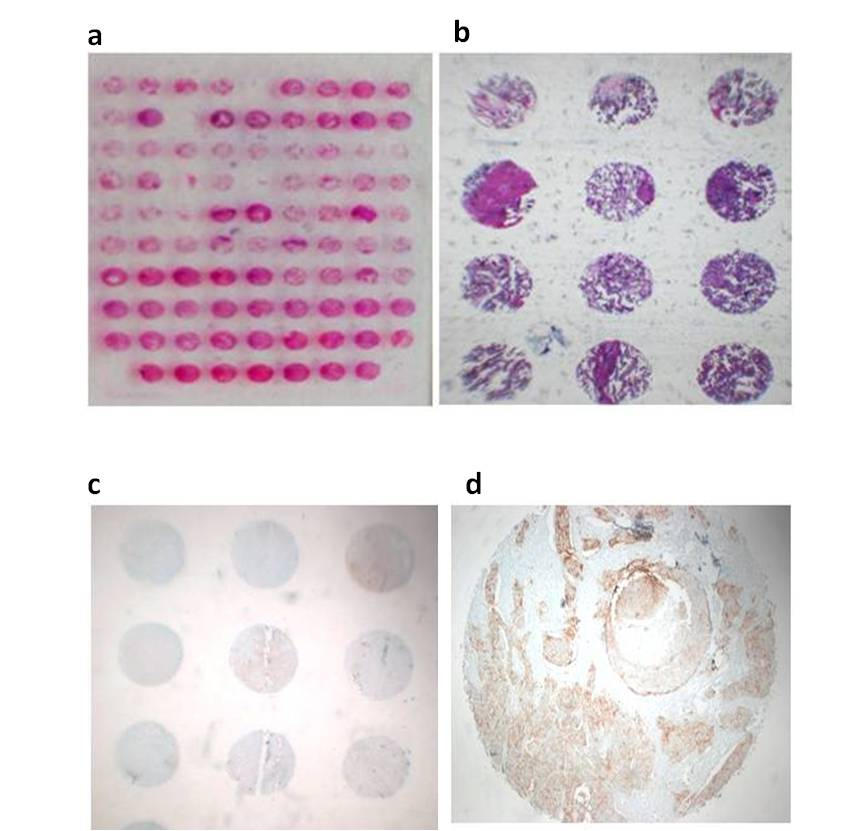

Supplement: Supplementary file 4 — Figure S2. Representative tissue microarray sections. (A) Images of the Hematoxylin & Eosin‐stained sections of a complete block (90 cores) and at a higher magnification, (B) immunohistochemistry staining of integrin αvβ6 on multiple TMA cores and a complete 1.5 mm core (1.75 sq. mm). TMA cores with less than 100 invasive tumor cells were considered inadequate for interpretation. A total of 189 tumor samples from the KMIO‐CS were used for building the TMA for αvβ6 IHC staining and among them 147 were interpretable. [file CAM4-5-2000-s004.tif]

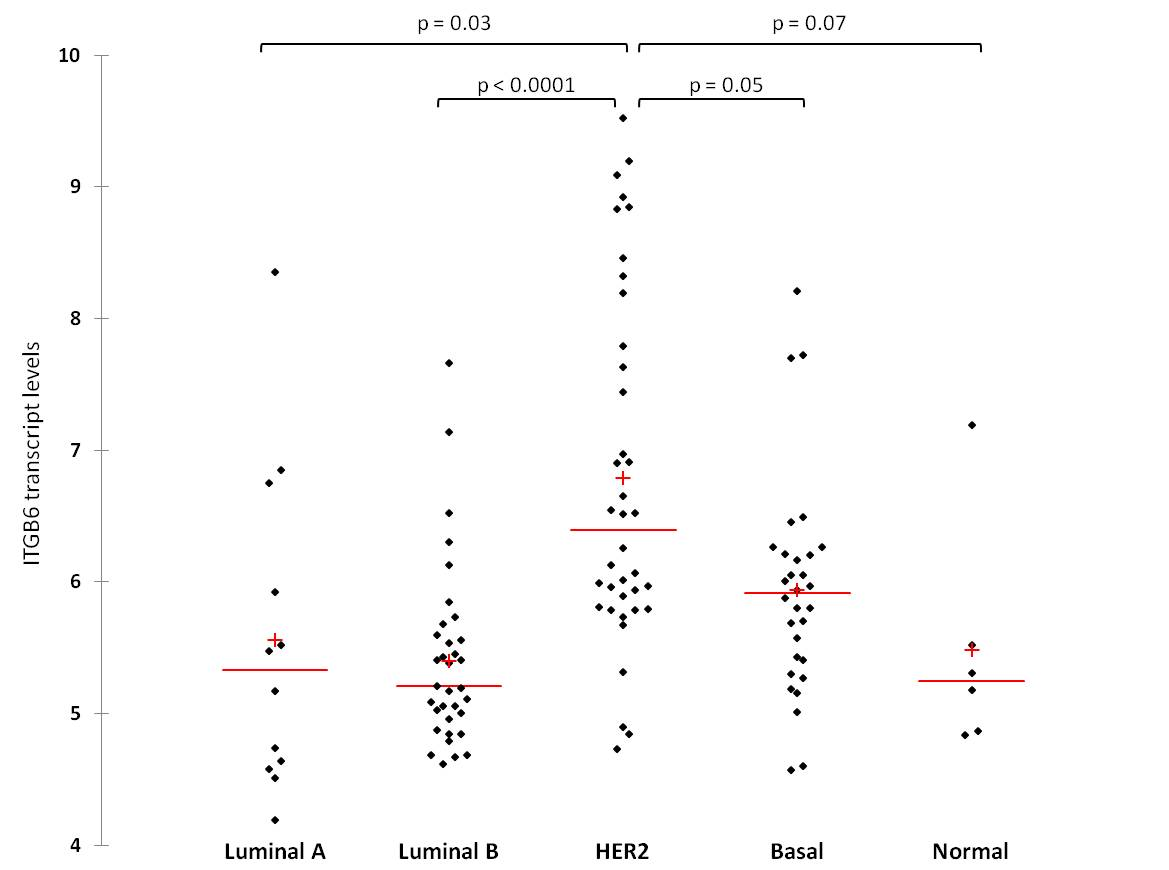

Supplement: Supplementary file 5 — Figure S3. In‐silico data analysis from the TEX trial dataset. The distribution of ITGB6 transcripts was plotted across the PAM50 subtypes and intergroup variability was analyzed using the Kruskal–Wallis test. A P‐value of <0.05 was considered significant. [file CAM4-5-2000-s005.tif]

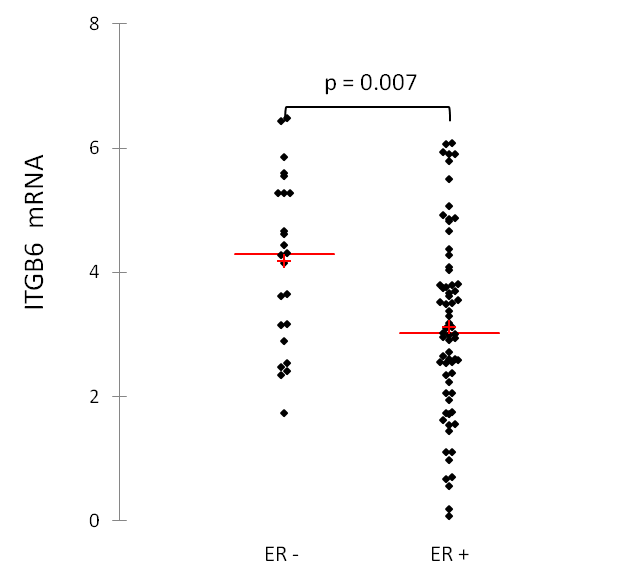

Supplement: Supplementary file 6 — Figure S4. Data analyzed from 93 HER2+ tumors from TCGA dataset with ITGB6 mRNA information. ITGB6 mRNA distribution between ER− and ER+ groups, a P‐value of <0.05 was considered statistically significant. [file CAM4-5-2000-s006.tif]

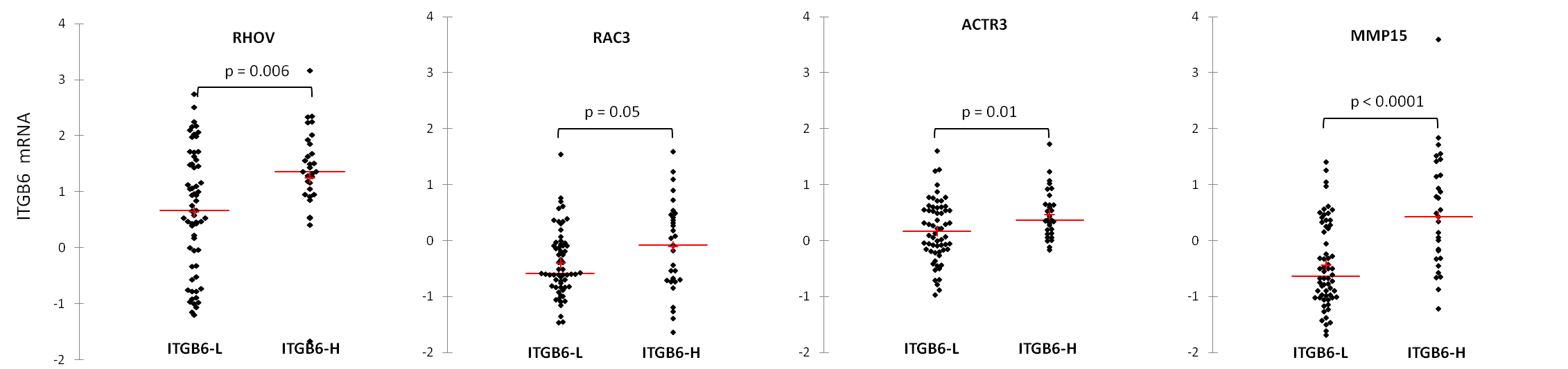

Supplement: Supplementary file 7 — Figure S5. Data analyzed from 93 HER2+ tumors from TCGA dataset with mRNA information available. Distribution of RHOV, RAC3, and MMP15 between ITGB6‐L and –H groups. [file CAM4-5-2000-s007.tif]

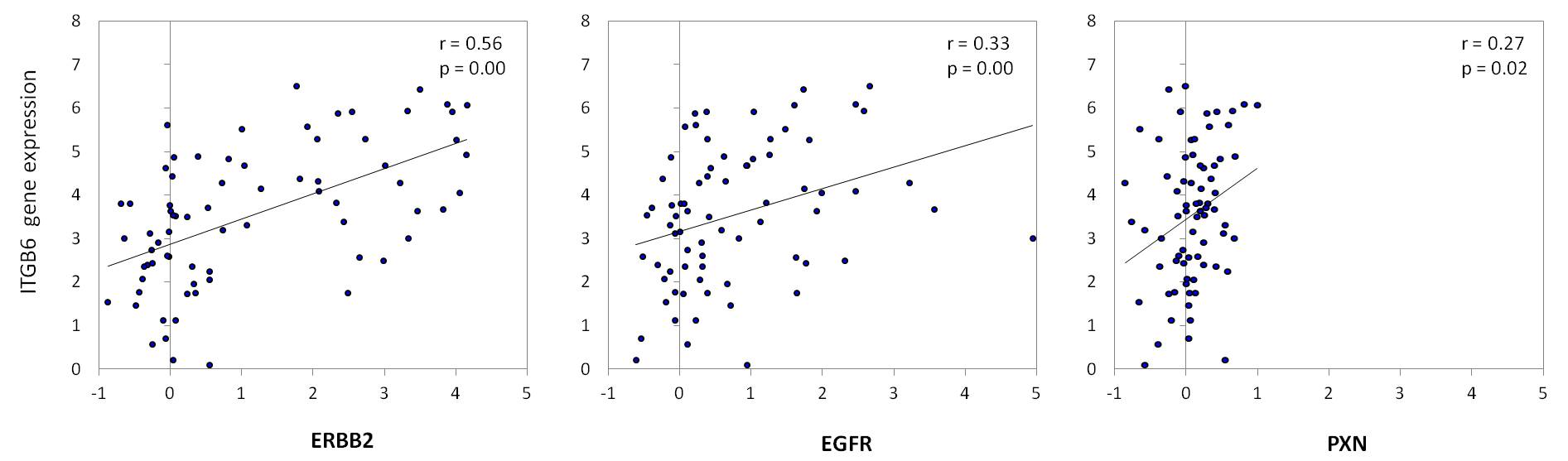

Supplement: Supplementary file 8 — Figure S6. Data analyzed from 73 HER2+ tumors from TCGA dataset with both ITGB6 mRNA and RPPA data available. Correlation plots between ITGB6 mRNA and ERBB2, EGFR, and PXN proteins. Pearson's correlation coefficient, r indicates the strength of correlation and a P‐value of <0.05 was considered statistically significant. [file CAM4-5-2000-s008.tif]
