# Supplementary material for: High expression of integrin β6 in association with the Rho–Rac pathway identifies a poor prognostic subgroup within HER2 amplified breast cancers
Source: Cancer Med. 2016 May 17;5(8):2000–11. doi: 10.1002/cam4.756 (PMC4873607; doi:10.1002/cam4.756)
Supplement: Supplementary file 9 [file CAM4-5-2000-s009.doc]

**Supplementary data**

**Table S1.** Clinico-pathological characteristics of 446 and 269 patients in the Nadathur-CS

**Table S2.** Primer sequences used for Gene expression analysis.

**Figure S1.** Distribution of ITGB6 mRNA in the two case series – KMIO and Nadathur-CS. The dynamic ranges of ITGB6 ARNU in both the CS were similar and ranged from 0-14. ITGB6 mRNA expression followed a normal distribution in both the case series.

**Figure S2**. Representative Tissue Microarray sections. a. Images of the Haematoxylin & Eosin stained sections of a complete block (90 cores) and at a higher magnification, b. Immunohistochemistry staining of integrin αvβ6 on multiple TMA cores and a complete 1.5 mm core (1.75 sq. mm). TMA cores with less than 100 invasive tumor cells were considered inadequate for interpretation. A total of 189 tumor samples from the KMIO-CS were used for building the TMA for αvβ6 IHC staining and among them 147 were interpretable.

**Treatment history of patients of the Nadathur-CS –**

Only 4/52 HER2+ patients received trastuzumab. This is in keeping with national trends of only a microscopic minority of HER2+ patients being able to afford trastuzumab. ~85% of the patients received appropriate complete standard-of-care multi-modal therapy including surgery, radiotherapy, chemotherapy and anti-hormonal therapy. Surgically, 22 patients were treated with BCS Breast Conservation Surgery (BCS) and 30 received either a MRM (Modified Radical Mastectomy) or total mastectomy. 7 patients did not receive appropriate complete systemic therapy for a variety of reasons (economic, advanced age, medical contraindications and defaulters for other reasons). Of the 45 who received chemotherapy, 30 received regimens containing both an anthracycline and a taxane of which 4 patients also received Herceptin; 15 were treated with anthracycline/cyclophosphamide/5-fluorouracil.

Of the 250 patients (non-metastatic at presentation) with complete clinical data examined for survival analysis approximately 20% (52/250) have had either a local and/or a distant recurrence at a median follow-up of 59 months. While most of these events occurred in patients who had received complete standard of care, 30% (16/52) patients with events had not completed their chemotherapy regimens for a variety of reasons including, advanced age, medical complications, economic constraints and people who refused chemotherapy for unstated reasons.

**Figure S3.** In-silico data analysis from the TEX trial dataset. The distribution of ITGB6 transcripts was plotted across the PAM50 subtypes and inter-group variability was analyzed using the Kruskal-Wallis test. A p-value of <0.05 was considered significant.

We analyzed the data from the Swedish multicenter trial – TEX (<http://www.clinicaltrials.gov/ct2/show/NCT01433614>) which included 111 patients with at least one biopsy from a confirmed loco-regional or distant breast cancer metastasis diagnosed between December 2002 and June 2007. All the patients had complete clinical details, follow-up information and gene expression information (Affymetrix array GPL10379). The gene expression microarray data was obtained from Gene Expression Omnibus under the accession number, GSE56493.

**TCGA data analysis –**

For the analysis of human breast cancer patient data to validate the distribution of ITGB6 mRNA between the Estrogen Receptor groups within the HER2+ subtype with a larger N=93 and to investigate the correlation between ITGB6 mRNA (Microarray), Rho-Rac pathway genes and other proteins (Reverse Phase Protein Array, RPPA), the data was downloaded from the data portal on TCGA website (https://tcga-data.nci.nih.gov/tcga/) and were TCGA level 3 data, the most highly processed data (https://tcga-data.nci.nih.gov/tcga/tcgaDataType.jsp). Pearson’s correlation coefficient (r) test was used to analyze correlation. We performed a HER2+ subtype specific analysis by deriving the data from TCGA Nature 2012 dataset comprising of 93 samples with mRNA information of which 73 samples had both mRNA and protein information.

**Figure S4.** Data analyzed from 93 HER2+ tumors from TCGA dataset with ITGB6 mRNA information. ITGB6 mRNA distribution between ER- and ER+ groups, a p-value of < 0.05 was considered statistically significant.

**Figure S5.** Data analyzed from 93 HER2+ tumors from TCGA dataset with mRNA information available. Distribution of RHOV, RAC3 and MMP15 between ITGB6-L and –H groups.

**Figure S6.** Data analyzed from 73 HER2+ tumors from TCGA dataset with both ITGB6 mRNA and RPPA data available. Correlation plots between ITGB6 mRNA and ERBB2, EGFR and PXN proteins. Pearson’s correlation coefficient, r indicates the strength of correlation and a p-value of < 0.05 was considered statistically significant.
